# Genetic Diversity, Extended-Spectrum Beta-Lactamase (ESBL) Screening, and Potential Public Health Implications of Gram-Negative Bacteria Recovered from Man-Made Lakes and Surrounding Vegetables

**DOI:** 10.3390/microorganisms13091997

**Published:** 2025-08-27

**Authors:** Ahou Cinthia Inès Yebouet, Kouakou Romain Fossou, Zaka Ghislaine Claude Kouadjo-Zézé, Anthony Ifeanyi Okoh, Adolphe Zézé

**Affiliations:** 1 Laboratoire de Microbiologie, Biotechnologies et Bioinformatique, Unité Mixte de Recherche et d’Innovation en Sciences Agronomiques et Procédés de Transformation, Institut National Polytechnique Félix Houphouët-Boigny, Yamoussoukro 1093, Côte d’Ivoire; cinthia.yebouet21@inphb.ci (A.C.I.Y.); romain.fossou@inphb.ci (K.R.F.); 2 Laboratoire Central de Biotechnologies, Centre National de la Recherche Agronomique, Abidjan 1740, Côte d’Ivoire; claudghis@gmail.com; 3 SAMRC Microbial Water Quality Monitoring Centre, University of Fort Hare, Alice 5700, South Africa

**Keywords:** Gram-negative bacteria, antibiotic resistance, water pollution, urban farming, One Health, Côte d’Ivoire

## Abstract

The emergence and dissemination of extended-spectrum beta-lactamase (ESBL)-producing bacteria pose a major public health threat, necessitating a One Health approach to addressing this threat. Thus, the diversity, ESBL production, and potential public health implications of Gram-negative bacteria recovered from man-made lakes and surrounding lettuce in Yamoussoukro, Côte d’Ivoire were assessed in this study. Also, the lakes’ physicochemical parameters were assessed and correlated with bacteria community using Pearson correlation. A total of 68 Gram-negative bacterial isolates were recovered from the samples and identified via *16S rDNA* gene sequencing. Phylogenetic analysis suggested multiple genus-/species-level variations within the isolates. *Escherichia coli* was the most prevalent in lake water (39.5%), while *Acinetobacter* was the dominant genus in lettuce (30%). *E. coli* isolates showed high resistance to ampicillin (90.9%), cefepime (72.7%), cefotaxime (68.2%), and aztreonam (63.6%). Moreover, ESBL production was confirmed in *E. coli* isolates (22.05%), predominantly mediated by the *bla_CTX-M_* gene. Multidrug-resistant phenotypes were widespread, yielding similar multiple antibiotic resistance index (MARI) values in water (0.27–0.63) and lettuce (0.27–0.81). These data indicate high environmental contamination, which unfortunately is not being taken into account by lettuce producers according to an interview. Statistical analyses showed a significant relationship between bacterial diversity and lakes’ physicochemical parameters, including dissolved oxygen, pH, and turbidity. The basic education level of farmers, the prevalence of ESBL-producing *E. coli*, and the high prevalence of MDR Gram-negative bacteria in both environmental and crop sources in Yamoussoukro underscore the need for both integrated surveillance and management strategies to mitigate potential microbial public health risks within a One Health framework.

## 1. Introduction

Gram-negative bacteria (GNB) constitute a vast and heterogeneous group of bacteria, distinguished by their complex cell wall structure, which includes an outer membrane that enhances their ability to survive in harsh environments and contributes to their resistance to various antibacterial agents [1,2]. Two major groups, *Enterobacteriaceae* (e.g., *Escherichia coli*, *Enterobacter* spp., *Proteus mirabilis*, and *Citrobacter* spp.) and non-fermenters, such as *Pseudomonas aeruginosa* and *Acinetobacter baumanii*, have been identified as environmental organisms, as well as opportunistic pathogens, with the potential to cause infections in humans and animals [3].

Currently, one of the most significant global health challenges is the emergence of multidrug-resistant (MDR) Gram-negative infections [3]. The intrinsic resistance mechanisms of these bacteria, in conjunction with their notable capacity for the acquisition and horizontal transfer of resistance genes via plasmids, transposons, and integrons, contribute to their emergence and dissemination as MDR strains [4]. The World Health Organization (WHO) has published a list of “priority pathogens,” which are increasingly difficult to treat [5]. Many of these pathogens are found to harbor an extended-spectrum beta-lactamase (ESBL) resistance mechanism [5]. Extended-spectrum beta-lactamases, primarily CTX-M, TEM, and SHV, are enzymes that facilitate the hydrolysis of extended-spectrum cephalosporins [6]. Of this family of enzymes, the most prevalent are CTX-M and its variations. As evidenced in more recent studies conducted in South Asia, 33% of *E. coli* isolates produce ESBL, with the CTX-M gene implicated in 58% of cases [7]. In Ethiopia, 18% of *Enterobacteriaceae* isolates were identified as ESBL producers [8]. Initially, ESBL outbreaks were primarily associated with hospitals [9]. However, they have since been identified in a range of settings, including water sources.

The role of water as a conduit for the dissemination of MDR bacterial strains has gained significant recognition in recent years [10,11]. The release of antibiotics into wastewater increases selective pressure on bacteria, thereby facilitating the proliferation of antibiotic resistance [12]. Furthermore, wastewater treatment processes are not entirely effective in eliminating microbial contaminants [13]. Consequently, wastewater is frequently discharged into receiving water sources, which allows resistant Gram-negative bacteria and genes encoding antibiotic resistance to reach agricultural soils and waterbodies [14]. For example, the prevalence of ESBL-*Enterobacteriaceae* in wastewater was estimated to be 24.81% [13]. In regions where untreated water from lakes or rivers is used for irrigation purposes, the transfer of resistant bacteria to vegetables can occur, thereby creating another pathway for human exposure to MDR bacteria [15,16].

Lettuce, a common ready-to-eat vegetable, plays a critical role in transmitting bacteria from the environment to humans, especially when grown in fields irrigated with contaminated water. More importantly, the presence of ESBL-producing bacteria on fresh produce like lettuce is especially concerning due to the potential for these pathogens to be ingested directly and for resistance genes to spread to the human microbiota [17,18]. Studies have shown that ESBL-*E. coli* can survive on lettuce for up to a week after contamination, highlighting the importance of food safety measures to prevent its spread [19].

Man-made lakes in Yamoussoukro, central Côte d’Ivoire, constitute a significant source of irrigation water for nearby vegetable fields, including lettuce [20]. Preliminary studies have revealed poor water quality and high levels of *E. coli*, with resistance to multiple antibiotics, including ampicillin, tetracycline, ciprofloxacin, and sulfamethoxazole [21,22]. In another study, *E. coli* was found on crops irrigated with the lake water [23]. However, there is a lack of information on the specific resistomes and the overall diversity of Gram-negative bacteria present in the lakes and on the crops. Given the growing concern about the role of contaminated environments in the spread of antibiotic-resistant bacteria, it is essential to monitor the presence and diversity of Gram-negative bacteria in water sources and on edible crops. This study focuses on Gram-negative bacteria isolated from man-made lakes in Yamoussoukro, Côte d’Ivoire, as well as the surrounding lettuce fields, which are regularly irrigated with water from the lakes. The goal was to assess the characteristics of these environmental bacteria by examining their diversity, resistance profiles, and multidrug resistance (MDR) potential, with a specific focus on ESBL production and related potential public health implications.

## 2. Materials and Methods

### 2.1. Study Area and Sample Collection

**Figure 1:**
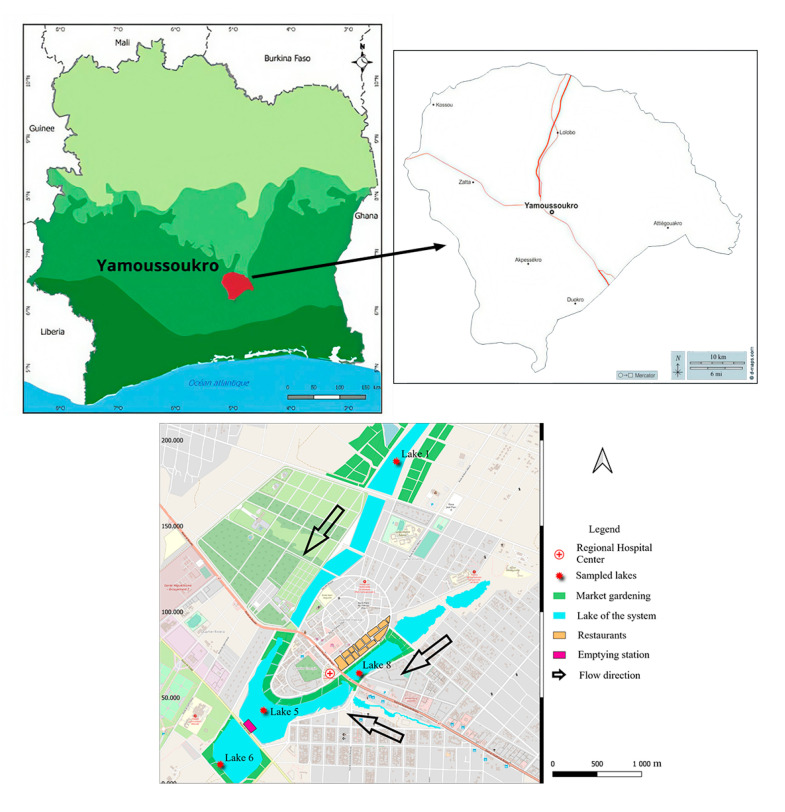
Yamoussoukro Lake system and location of study target lakes.

This study has been carried out in Yamoussoukro, in central Côte d’Ivoire (6°40′/7°00′ N and 5°10′/5°20′ W) (Figure 1). This city was created with a system of ten man-made lakes and has been established as the political capital of Côte d’Ivoire since 1983. Connected by gravity, these lakes are an important part of the city’s tourist attractions and cultural heritage. Various human activities are carried out around these lakes, including gardening, livestock farming, restaurant operation, etc. For this study, four lakes within the Yamoussoukro Lake system, which have been previously studied with regard to their microbiological quality [21], were selected. The selected lakes were distributed throughout the system: lakes 1 and 8 are located upstream, at the entrance to the two branches of the system; lake 5 is located in the center of the system; and lake 6 is situated downstream of the system (Figure 1). Sampling was conducted over two dry periods: from November 2021 to January 2022 and from September 2022 to November 2022. For each lake, 1000 mL of water was collected in a U-shaped pattern at a depth of 50 cm [24] at three sampling points. A total of 15 water samples were collected for each lake. In consideration of the vegetable crops cultivated in the vicinity of the lakes, lettuce (*Lactuca sativa*) was identified as a suitable subject for study. A total of eight lettuce plants exhibiting no apparent deterioration or visible cracking were randomly selected within the proximity of each lake and placed in sterile freezer bags. All the collected samples were placed in a cooler at a temperature of 4 °C and transferred to the laboratory as quickly as possible for further analysis.

### 2.2. Survey of Agricultural Producers

The survey was conducted using a structured questionnaire designed on the KoboToolbox platform and administered during face-to-face interviews with consenting farmers (questionnaire available as follows: https://kf.kobotoolbox.org/#/forms/a7NeSfdb4gbSmwexWfmePo, accessed on the 15 February 2024). The objective was to investigate farming practices that may potentially contribute to the microbiological contamination of lettuce crops by Gram-negative MDR bacteria and the extent to which these producers are aware of the microbiological risks. Thus, a total of 65 lettuce growers were surveyed. The questionnaire encompassed several key themes, including sociodemographic data, irrigation water use, agricultural input practices, and post-harvest hygiene and management.

### 2.3. Physicochemical Analysis of Lake Water

The physicochemical parameters of the lake water were analyzed in order to characterize the environmental conditions that may influence the survival and proliferation of Gram-negative bacteria. The following parameters were measured: pH, temperature, redox potential (mV), turbidity (NTU), conductivity (µS/cm), dissolved oxygen (mgO_2_/L), total dissolved solids (TDS, mg/L), total hardness (TH, mg/L), nitrate (NO_3_^−^, mg/L), nitrite (NO_2_^−^, mg/L), phosphate (PO_4_^3−^, mg/L), ammonium (NH_4_^+^, mg/L), bicarbonate (HCO_3_^−^, mg/L), calcium (Ca_2_^+^, mg/L), and magnesium (Mg_2_^+^, mg/L) concentrations. The analyses were conducted in accordance with the standard Afnor methods [25]. The measurements were taken using a multifunctional pH meter (PCE-PHD 1, PCE Instruments, Meschede, Germany) and a spectrophotometer (Jasco V-530 UV/VIS, JASCO Corporation, Japan).

### 2.4. Isolation of Gram-Negative Bacteria

Prior to isolation, 1 mL of each water sample and 2.5 g of each lettuce sample were enriched in 9 mL of Tryptone Soy Broth (Merck, Darmstadt, Germany) and 22.5 mL of Peptone Water (Oxoid, Basingstoke, UK), respectively, and incubated at 37 °C for four hours. Following the incubation, a three-series ten-fold dilution of each pre-enriched culture was conducted (10^−1^, 10^−2^, and 10^−3^). Subsequently, 100 µL of each prepared dilution, in addition to the pre-enrichment suspensions, were plated in duplicates on distinct media, including Chromocult culture agar (Merck, Darmstadt, Germany), *Salmonella*-*Shigella* agar (Oxoid, Basingstoke, UK), and *Pseudomonas* base agar supplemented with CFC selective supplement (ISO) (Oxoid, Basingstoke, UK). These media are commonly recommended for the isolation of Gram-negative bacilli. To maximize the isolation of resistant strains, all media were also supplemented with 2 mg/mL Ceftazidime. The plates were then incubated at 37 °C for 24 h. When isolates started growing, a sample was restreaked on agar-based medium to isolate single colonies. Thus, all of the isolates described in this study were purified, and a Gram stain was conducted to confirm the Gram type prior to subsequent molecular analyses. In all these analyses, *Pseudomonas aeruginosa* ATCC 27853 and *Escherichia coli* ATCC 8739 were used as reference strains.

### 2.5. Molecular Identification of the Isolates

Genomic DNA of each isolate was extracted from pure colonies following the standard CTAB method [26]. DNA was extracted from colonies that had been grown for 18–24 h, and 5 µL of DNA template was used for PCR assays. Additionally, the universal primers 27F (AGA GTT TGA TCM TGG CTC AG) and 1492R (TAC GGY TAC CTT GTT ACG ACT T) were utilized to amplify the *16S rDNA* gene.

The PCR reaction mixture was prepared, resulting in a final volume of 50 µL, comprising 25 µL of OneTaq^®^ Quick-Load^®^ 2X Master Mix with Standard Buffer (New England Biolabs (NEB), Ipswich, MA, USA), 5 µL each of forward and reverse primer (manufactured by Eurofins, Luxembourg, Luxembourg), 5 µL of the DNA template, and sterile distilled water to reach a total volume of 50 µL. The amplifications were conducted using a iCycler™ thermal cycler (Bio-Rad, Hercules, CA, USA), with the following cycling parameters: an initial denaturation at 95 °C for 2 min, followed by 30 cycles of denaturation at 95 °C for 30 s, annealing at 55 °C for 30 s, 72 °C for 1 min 30 s for cycling extension, and a final extension at 72 °C for 2 min. Subsequently, 6 µL of the amplicons were resolved in a 1% agarose gel (Benchmark Scientific, Sayreville, NJ, USA) stained with SafeView (Applied Biological Materials Inc. (ABM), Richmond, BC, Canada)), including a 1 kb DNA ladder (New England Biolabs (NEB), Ipswich, MA, USA, which served as a molecular size standard. The electrophoresis was conducted at 50 volts for 30 min, and the resulting gels were visualized using the UVP Photodoc-it imaging system (Analytik Jena, Jena, Germany). Genomic DNA of *P. aeruginosa* ATCC 27853 and *E. coli* ATCC 8739 were utilized as positive controls, while a microtube containing only the DNA-free reaction medium served as the negative control.

### 2.6. 16S rDNA Sequencing and Phylogenetic Analysis

The PCR product was purified and sequenced using the Sanger method (Inqaba, Pretoria, South Africa). The sequences were then assembled into double-stranded DNA using the BioEdit version 7.25 program and manually edited when necessary. Edited sequences were then compared with those already deposited in GenBank using the BLAST algorithm (NCBI; https://blast.ncbi.nlm.nih.gov, accessed on 25 March 2025) to identify our isolates and find their closely related reference strains. Accession numbers of the isolates are provided in Table S2.

Prior to the phylogenetic reconstruction, a multiple sequence alignment was conducted by aligning the consensus sequences of the *16S rDNA* genes with those of the reference type strains collected from GenBank. In brief, the *16S rDNA* edited sequences were aligned with MUSCLE as implemented in MEGA software version 11, and the best-fit nucleotide substitution model was selected according to the Bayesian information criterion (BIC) [27]. A subsequent phylogenetic tree was reconstructed using the neighbor-joining (NJ) and maximum likelihood (ML) methods [28,29], with a bootstrap value of 1000 replicates [30]. The resulting tree was visualized with iTool [31].

### 2.7. Antibiotic Susceptibility Testing

The antibiotic resistance profiles of the identified isolates were analyzed using the standard Kirby–Bauer disc diffusion tests [32], with some modifications. The colonies were selected from 24 h pure cultures, emulsified in sterile 0.9% NaCl solution, and adjusted to match the 0.5 McFarland standard. The mixture was evenly inoculated on Mueller–Hinton agar by flooding the entire surface with the inoculum, and the excess liquid was then carefully removed using a sterile pipette. Antibiotic discs were subsequently added to the agar using sterile forceps. The plates were incubated for 24 h at 37 °C. The inhibition zones were measured in millimeters and classified as susceptible (S), intermediate (I), or resistant (R) according to the standards recommended by the European Committee on Antimicrobial Susceptibility Testing [33]. The breakpoint was displayed in Table S1. Antibiotics included in the analysis were gentamycin (CN-10 µg), ampicillin (AMP-10 µg), amoxicillin–clavulanic acid (AMC-20/10 µg), ticarcillin–clavulanic acid (TIM-75/10 µg), imipenem (IMP-10 µg), cefotaxime (CTX-30 µg), cefepime (FEP-30 µg), ceftazidime (CAZ-30 µg), ciprofloxacin (CIP-5 µg), cefoxitin (FOX-30 µg), chloramphenicol (C-30 µg), and aztreonam (ATM-30 µg) (Oxoid, Basingstoke, UK). Concurrently, the double synergy test was conducted by placing discs of amoxicillin + clavulanic acid or ticarcillin + clavulanic acid 2.5 cm apart [34,35]. Isolate was identified as an ESBL producer when a “champagne cork” synergy was observed. *Escherichia coli* ATCC 8739 and an in-house ESBL-*E. coli* strain were utilized as reference strains for the negative and positive controls.

### 2.8. Evaluation of Multiple Antibiotic Resistance Phenotypes (MARPs) and the Multiple Antibiotic Resistance Index (MARI)

The resistance of isolates against three or more antibiotics was used as a criterion to identify MDR strains [36]. Subsequently, the multiple antibiotic-resistant phenotypes (MARPs) were assessed for each MDR isolate, and its multiple antibiotic resistance index (MARI) was then generated using the following mathematical equation [37]:

MAR index=a/b

where “a” denotes the number of antimicrobial agents to which the isolates exhibit resistance to and “b” indicates the total number of antimicrobial agents against which each isolate was tested. MARI that is equal to or greater than 0.2 shows that antimicrobial agents are intensively used in that area, thus promoting the emergence of antibiotic resistance [37,38].

### 2.9. Screening for ESBL Resistance Genes

Following the double synergy test, the isolates that exhibited the “champagne cork” synergy were subjected to screening for the presence of three common ESBL antibiotic resistance encoding genes *(bla_TEM_*, *bla_CTX-M_*, and *bla_SHV_*) using the PCR technique. The primer sequences, as well as their amplicon size and cycling conditions, are listed in Table 1. *E. coli* ATCC 8739 and an in-house ESBL-*E. coli* strain were also used as reference strains.

### 2.10. Data Analysis

Data treatment and statistical analysis were performed using Microsoft Excel version 2016 (Microsoft Ltd., Redmond, WA, USA) and Rstudio version 4.2.2 (R Core Team, Vienna, Austria) [42]. Normal distribution tests were performed using the Shapiro–Wilk test, while the Bartlett test was used to assess variance homogeneity. When the two assumptions are not met, e.g., for the lake’s physico-chemical properties, we used the Kruskal–Wallis test, followed by a Dunn a posteriori test performed with the “dunn.test” package version 1.3.6. Tukey’s post hoc test (emmeans package version 1.11.2) was applied for pairwise comparisons of means. Bacterial community diversity was assessed on the basis of calculations using Shannon–Wiener (H′) ecological indices [43] with the vegan package version 2.7.1. Relationships between lake properties and bacterial abundance and diversity were assessed using Pearson correlation with the stats package version 4.5. All graphs were generated using the “ggplot2” package version 3.5.2 [44]. All data sets on bacterial abundance and lake chemical properties were subjected to principal component analysis (PCA) using the “FactoMineR” (version 2.11) and “factoextra” (version 1.0.7) packages. The relevance of the data for PCA was verified using the Kaiser–Meyer–Olkin (KMO) index. PCA was used to explore the associations between response variables, i.e., chemical properties regarding lakes and bacterial communities.

## 3. Results

### 3.1. Sociodemographic Characteristics of Lettuce Producers Around Yamoussoukro Lakes

The survey identified several trends among the interviewed producers (Table 2). About 60% of them were male and between 30 and 45 years old. In terms of farming experience, 35.38% of respondents indicated that they had between 5 and 10 years of experience in market gardening. Furthermore, 81.53% of the farmers were Ivorian. Finally, the level of education was generally low, with 73.84% of growers reporting that they were illiterate, as displayed in Table 2.

### 3.2. Biocontamination Risk Practices for Lettuce in the Field

**Figure 2:**
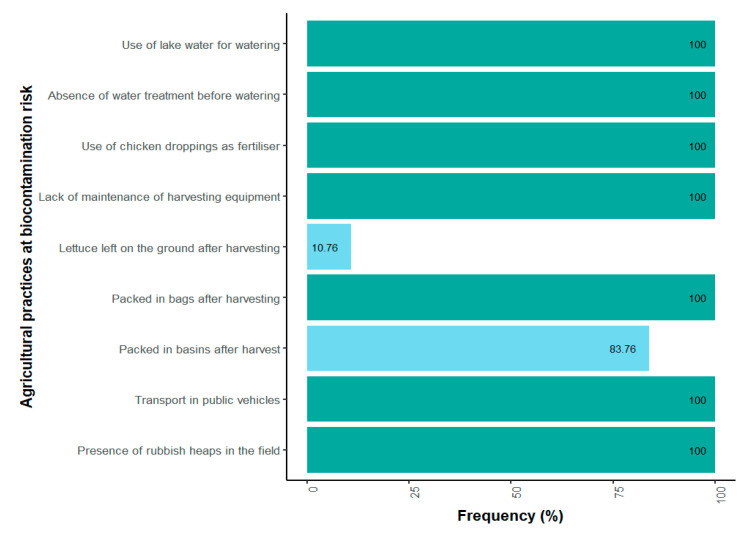
Frequency of biocontamination risk practices in lettuce fields.

**Figure 3:**
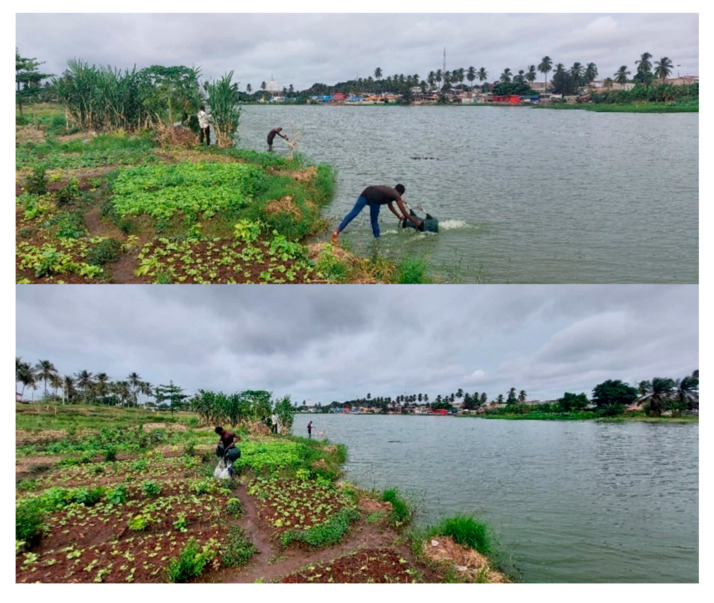
View of lettuce crops irrigated with lake water.

Farmers’ daily practices illustrate a pathway toward biocontamination risks (Figure 2). Every day, fields are irrigated exclusively with lake water, used without any prior treatment (Figure 3). All the farmers use chicken manure as their only organic fertilizer, and harvesting equipment is not washed after use. During harvesting, different packaging methods are used. All of the producers use bags to pack the lettuce. Furthermore, 83.07% of them utilize basins, while 10.76% of them place the lettuce directly on the ground. The lettuce harvested in this way is primarily sold at local markets in Yamoussoukro. However, 90.76% of producers indicated that their products are also shipped to other cities, mainly the economic capital, Abidjan. All producers usually consume a part of their own products. In terms of risk perception, 100% of the farmers said they were aware of food-borne infections. However, none of them were aware of antibiotic resistance at the time of the survey.

### 3.3. Physicochemical Properties of the Lakes

Significant differences (*p*-value < 0.05) in the water’s physicochemical properties were observed among the four lakes (Table 3). The total dissolved solids (TDS) levels vary significantly among the lakes, with the highest recorded in lake 5 (2.25 ± 0.06 mg/L) and the lowest recorded in lake 1 (1.44 ± 0.49 mg/L). Conductivity levels differed significantly between lakes, with lake 6 (3.77 ± 0.1 µS/cm) having the highest conductivity and lake 1 (2.41 ± 0.82 µS/cm) having the lowest. Ammonium (NH_4_^+^) and bicarbonate (HCO_3_^−^) levels were significantly higher in lake 5 (11.82 ± 1.2 and 334 ± 20.9 mg/L, respectively) and lower in lake 1 (0.65 ± 0.41 and 142 ± 50.2 mg/L, respectively). Lake 6 has a high TH with a higher calcium concentration (0.67 ± 0.02 mg/L). Turbidity was significantly higher in lake 8 (216.67 ± 52.08) compared to lake 1, lake 5, and lake 6 (*p* < 0.001). Lake 8’s water is much more alkaline, with a pH of 8.47 ± 0.55, unlike lake 1, which has a pH that tends towards acidity. The lake temperatures ranged from 22 to 25 °C, with the highest temperature recorded in lake 5 (25.2 ± 0.61). Statistical analysis revealed no significant differences (*p*-value > 0.05) among the lakes in terms of NO_3_^−^, NO_2_^−^, PO_4_^3−^, Mg_2_^+^, and dissolved oxygen values.

### 3.4. Distribution and Diversity of the Bacterial Community in Each Sample

**Figure 4:**
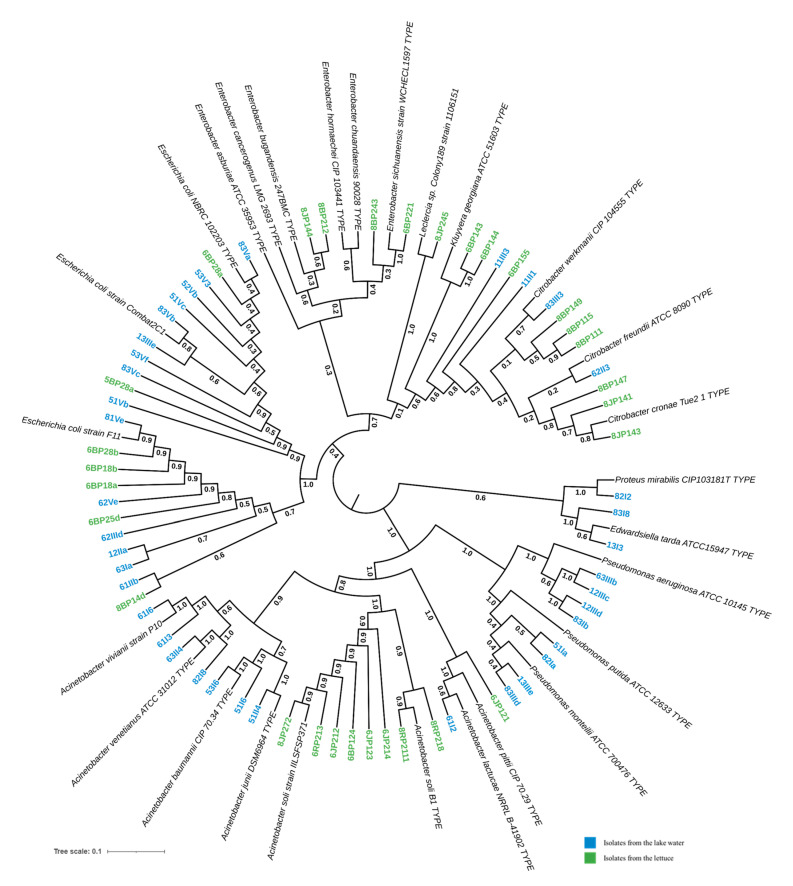
Phylogenetic tree of potential pathogenic bacteria isolated from lake waters and lettuce in Yamoussoukro, Côte d’Ivoire, based on *16S rDNA* gene sequences. The sequences of the best match reference type strains were collected from GenBank and then aligned using MUSCLE, as implemented in MEGA software version 11, with the isolates’ *16S rDNA* edited sequences. A subsequent phylogenetic tree was reconstructed using the neighbor-joining (NJ) and maximum likelihood (ML) methods with a bootstrap value of 1000 replicates. The resulting tree was visualized with iTool.

A total of 68 isolates were identified via *16S rDNA* sequencing, including 38 from the lake water and 30 from the lettuce. Phylogenetic analysis suggested that they belonged to nine different taxa, with *E. coli* being the major isolates (>30%) (Figure 4). These taxa were identified at the genus and/or species level, including *Escherichia coli* (32.35%, *n* = 22), *Acinetobacter* spp. (25.37%, *n* = 17), *Citrobacter* spp. (16.17%, *n* = 11), *Pseudomonas* spp. (11.94%, *n* = 8), *Enterobacter* spp. (5.97%, *n* = 4), *Kluyvera georgiana* (2.98%, *n* = 2), *Edwardsiella tarda* (2.98%, *n* = 2), *Proteus mirabilis* (1.49%, *n* = 1), and *Leclercia* spp. (1.49%, *n* = 1).

**Figure 5:**
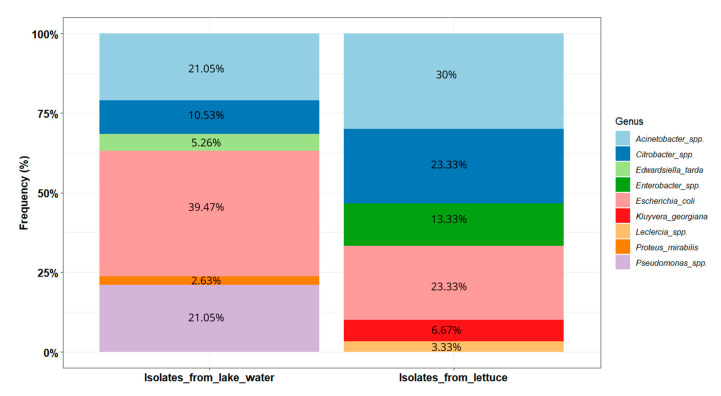
Distribution of the potential pathogenic bacteria identified in lake waters and lettuce in Yamoussoukro, Côte d’Ivoire.

The Shannon diversity index values were 1.51 and 1.44, respectively, for the lake and lettuce samples. The distribution of the isolates within these two types of environments is presented in Figure 5. In the lake water samples (*n* = 38), *E. coli* was the most prevalent (39.47%, *n* = 15). *Acinetobacter* isolates were the second most prevalent, pertaining to *Acinetobacter baumannii* (*n* = 2), *Acinetobacter venetianus* (*n* = 2), *Acinetobacter vivianii* (*n* = 2), *Acinetobacter junii* (*n* = 1), and *Acinetobacter lactucae* (*n* = 1). *Pseudomonas* isolates were closely related to *Pseudomonas aeruginosa* (*n* = 4), *Pseudomonas putida* (*n* = 2), and *Pseudomonas monteilii* (*n* = 2). The *Citrobacter* isolates are closely related to *Citrobacter werkmanii* (*n* = 1) and *Citrobacter freundii* (*n* = 1) (Figure 4).

In the lettuce samples (*n* = 30), the most dominant genus was *Acinetobacter* (30%, *n* = 9). *Acinetobacter* isolates are likely to be *Acinetobacter soli* (*n* = 8) and *Acinetobacter pittii* (*n* = 1). *Enterobacter* isolates potentially belong to species *Enterobacter bugandensis* (*n* = 2) and *Enterobacter sichuanensis* (*n* = 2). The *Citrobacter* isolates were closely related to *Citrobacter werkmanii* (*n* = 3), *Citrobacter cronae* (*n* = 3), and *Citrobacter* sp. (*n* = 1).

### 3.5. Relationship Between Lake Physicochemical Properties and Bacterial Diversity and Abundance

The Pearson correlation coefficients among bacterial abundance, the Shannon diversity index, and the lakes’ physicochemical properties are shown in Table 4. A significant negative correlation (*p* < 0.05) was observed between the Shannon diversity index and dissolved oxygen (*r* = −0.99). In contrast, non-significant negative correlations were detected among the Shannon diversity index, ammonium level (*r* = −0.64), and calcium level (*r* = −0.52). In addition, significant positive correlations (*p* < 0.05) were detected among bacterial abundance, turbidity (*r* = 0.962), and pH (*r* = 0.95). Non-significant positive correlations were also observed among bacterial abundance, TDS (*r* = 0.58), conductivity (*r* = 0.542), total hardness (*r* = 0.63), magnesium level (*r* = 0.85), temperature (*r* = 0.61), and potential redox (*r* = 0.78)

**Figure 6:**
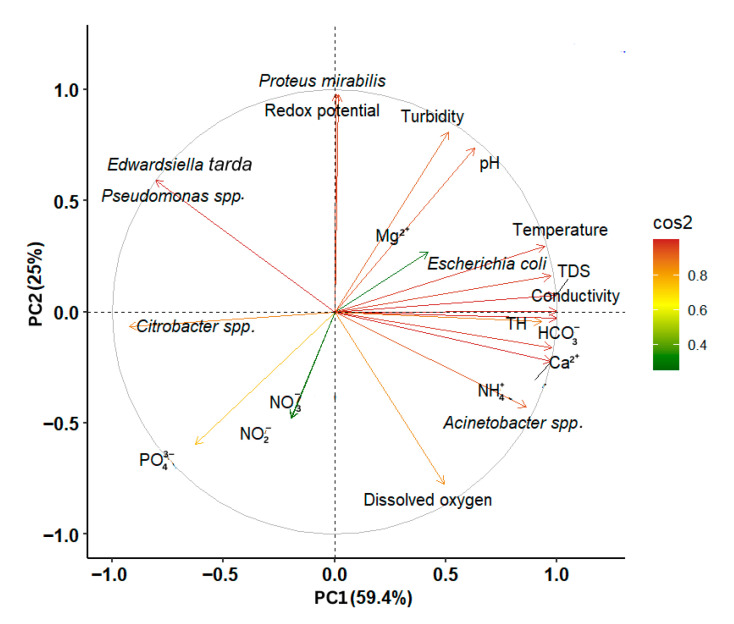
Principal component analysis (PCA) examining the distribution of chemical properties in lakes correlated with the bacterial community. The colored arrows represent the active variables (standardized physicochemical properties and bacterial communities) used to construct the PCA.

Principal component analysis (PCA) was performed to show the relationships between the lakes’ physicochemical properties and bacterial community composition. The first two principal components explained a total of 84.4% of the variance in the data, with PCA1 accounting for 59.4% and PCA2 accounting for 25% (Figure 6). The first axis (PCA1), which explained the largest portion of the variability, showed positive correlations with TDS (*r* = 0.99), conductivity (*r* = 0.99), NH_4_^+^ (*r* = 0.95), HCO_3_^−^ (*r* = 0.99), Ca_2_^+^ (*r* = 0.97), TH (*r* = 0.93), temperature (*r* = 0.96, pH (*r* = 0.67), dissolved oxygen (*r* = 0.44), and redox potential (*r* = 0.02). Lakes with higher values for these parameters tend to cluster on the positive side of PCA1. In contrast, the second axis (PCA2) showed a negative correlation with dissolved oxygen (*r* = −0.79), conductivity (*r* = −0.06), NH_4_^+^ (*r* = −0.22), HCO_3_^−^ (*r* = −0.02), and Ca_2_^+^ (*r* = −0.15).

Genera *Pseudomonas* spp., *Edwardsiella tarda*, *Escherichia coli*, and *Proteus mirabilis* are associated with higher levels of turbidity, pH, Mg_2_^+^, temperature, TDS, conductivity, and redox potential, as well as with lower levels of dissolved oxygen. *Citrobacter* spp. was linked to lower values of TDS, conductivity, NH_4_^+^, HCO_3_^−^, Ca_2_^+^, TH, and temperature. As for *Acinetobacter* spp., it showed an association with higher TDS, conductivity, NH_4_^+^, HCO_3_^−^, Ca_2_^+^, TH, and temperature, as well as lower dissolved oxygen levels.

### 3.6. Antibiotic Susceptibility Patterns

The results of the antibiogram were used to determine antibiotic resistance (Figure S1). Based on the total number of isolates, ampicillin showed the highest resistance rate (47%), followed by ceftazidime (35.29%) and cefepime (32.35%). Conversely, the antibiotics exhibiting the lowest resistance rates were chloramphenicol (2.9%) and ticarcillin/clavulanic acid, for which no resistance was observed.

**Figure 7:**
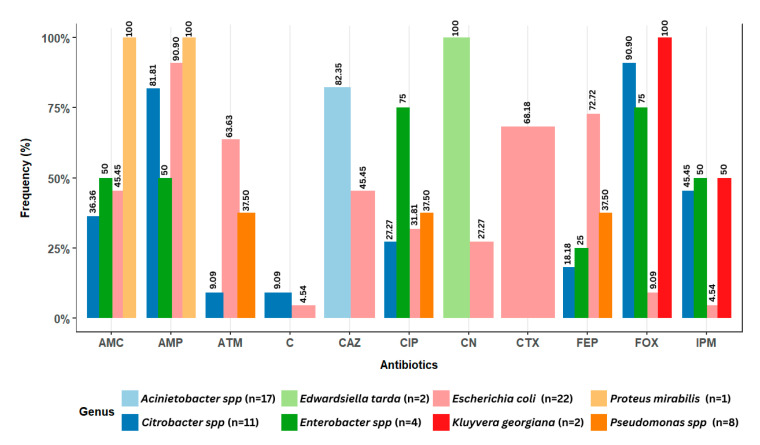
Antibiotic resistance distribution of potential pathogenic bacteria isolated from lake waters and lettuce in Yamoussoukro, Côte d’Ivoire. n represents the total number of isolates. AMP: ampicillin; CN: gentamicin; IPM: imipenem; FEP: cefepime; CIP: ciprofloxacin; FOX: cefoxitin; ATM: aztreonam; CTX: cefotaxime; C: chloramphenicol; AMC: amoxicillin/clavulanic acid; CAZ: ceftazidime.

More specifically, the distribution of antibiotic resistance for each species/genus isolated from the lake water and lettuce is presented in Figure 7. A significant proportion of *E. coli* isolates showed resistance to ampicillin (90.9%), cefepime (72.72%), cefotaxime (68.18%), and aztreonam (63.63%). For *Citrobacter* isolates, resistance to cefoxitin (90.9%) and ampicillin (81.81%) was observed. *Enterobacter* isolates demonstrated resistance to ciprofloxacin (75%) and cefoxitin (75%). Some *Pseudomonas* isolates showed resistance to cefepime, ciprofloxacin, and aztreonam at a frequency of 37.5% for each antibiotic. All *Kluyvera gergiana* isolates demonstrated 100% resistance to imipenem. *Acinetobacter* isolates showed resistance only to ceftazidime (82.35%). The bacteria *Edwardsiella tarda* and *Proteus mirabilis* were highly susceptible to antibiotics. However, 100% resistance was observed in *Edwardsiella tarda* to gentamicin, as well as in *Proteus mirabilis* to ampicillin (100%) and amoxicillin/clavulanic acid (100%). Finally, the *Leclercia* isolate exhibited no resistance to any of the tested antibiotics.

### 3.7. Prevalence and Antibiotic Resistance Genes Among the ESBL Bacteria

**Figure 8:**
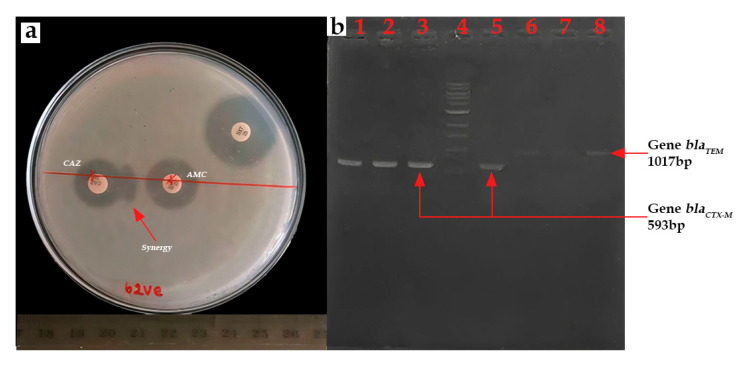
(**a**) Positive ESBL result to double synergy test and (**b**) PCR product for *bla_CTX-M_* and *bla_TEM_* gene (**b**) Lanes 1–3 and 5: *bla_CTX-M_*; Lane 4: 1kb DNA ladder; and Lanes 6–8: *bla_TEM_*.

β-lactamase production was confirmed by the synergistic effect observed between ceftazidime (30 μg) and amoxicillin/clavulanic acid (20 μg/10 μg) discs (Figure 8a). ESBL prevalence was found to be 22.05% (15/68). All isolates exhibiting this resistance phenotype belonged to the *E. coli* species. Ten strains were isolated from lake samples, while five were recovered from lettuce samples. All 15 ESBL-*E. coli* isolates were found to be positive for *bla_CTX-M_* gene presence (100%) (Figure 8b). The *bla_TEM_* gene was identified in 11 isolates (73.33%), occurring in conjunction with the *bla_CTX-M_* gene. The *bla_SHV_* gene was not detected.

### 3.8. MARI Index of the Lake and Lettuce Samples

Several isolates (38.23%, *n* = 26) were resistant to more than three classes of antibiotics and were classified as MDR strains. In lake water samples, *E. coli* and *Pseudomonas* spp. exhibited five and one MARP patterns, respectively, which ranged from three to seven. Many of these patterns were single. Only three patterns were observed two and four times, as illustrated in Table 5. In lettuce samples, *E. coli*, *Citrobacter* spp., and *Enterobacter* spp. exhibited six, five, and one MARP patterns, respectively ranging from three to nine. Many of these patterns were single, with only one occurring in duplicate. The MAR index (MARI) of all the MDR isolates recovered from lake water ranged from 0.27 to 0.63, and it ranged from 0.27 to 0.81 for those recovered from lettuce samples (Table 5).

## 4. Discussion

This study evaluated the genetic diversity, antibiotic resistance profiles, and potential public health implications of 68 Gram-negative bacteria recovered from lake water and lettuce in Yamoussoukro, Côte d’Ivoire, in addition to screening for ESBL production.

First, the physicochemical parameters of the lakes were assessed. The measured physicochemical parameters provide information on the quality of the lake waters. Water temperature and pH differed significantly among the lakes (*p* < 0.001). Lake 1 was the coolest (22.83 ± 0.55 °C), with a more acidic pH (5.96 ± 0.4), compared to lakes 5, 6, and 8, which were warmer, with a neutral to basic pH. Similarly, bicarbonate ion values differed statistically, with lake 1 showing a lower value (142 ± 50.2 mg/L) compared to the other lakes. These results are consistent with Anoman et al., 2019 [21], who also observed that lake 1 had a lower temperature and pH compared to the other lakes. However, the values remain within the normal range for water used for agriculture [45]. Dissolved oxygen concentrations varied from 4.47 ± 0.35 mgO_2_/L in lake 8 to 5.87 ± 1.19 mgO_2_/L in lake 5 and did not show statistically significant differences between the lakes. According to the guidelines, the required concentration of dissolved oxygen is 6 mg/L for drinking water and 4–6 mg/L for fish and domesticated animals [46,47]. Thus, the observed values indicate that the lakes are well oxygenated. Water hardness reflects the geological nature of the area with which the water has been in contact [48]. The major cations causing water hardness are Ca^2+^ and Mg^2+^. In this study, values obtained for Ca^2+^ ranged from 0.41 ± 0.04 mg/L (lake 1) to 0.67 ± 0.02 mg/L (lake 6) and differed statistically from one lake to another. The same is true of the TH value (range from 0.54 ± 0.13 mg/L to 0.9 ± 0.06 mg/L), while on the other hand, the values for Mg^2+^ are not statistically different (range from 0.13 ± 0.1 mg/L to 0.23 ± 0.05 mg/L). According to the classification system proposed by Sawyer et al. (2003) [49], which categorizes water as soft (<75 mg/L), moderately hard (75–150 mg/L), hard (150–300 mg/L), and very hard (>300 mg/L), the lake water can be classified as soft. Conductivity and TDS values were in agreement: very low, with statistically significant differences among the lakes. These two parameters are correlated and indicate the salinity level in water [50]. The values obtained in this study meet the permissible value for agricultural water [45]. Values for turbidity range from 7 ± 5.26 NTU to 216.67 ± 52.08 NTU, with statistically significant differences among the lakes. Except for lake 1, all the lakes exceed the permissible value [47,51]. Turbidity is a key water quality parameter for drinking water due to its link with pathogenic microorganisms. Thus, the indication is that waters with higher turbidity values could be sources of water-borne diseases [48]. Nitrate, nitrite, and phosphorus values did not show any statistically significant differences among the lakes, despite numerical variations, unlike the values for ammonium, for which the difference was significant (*p* < 0.001). The concentration of nutrients, primarily nitrogen and phosphorus compounds, can compromise water quality [52]. As a result, excessive nutrient input into an aquatic system leads to eutrophication, a biological response that results in adverse effects such as poor water quality, which in turn restricts its use in industry and agriculture [52,53]. These findings are consistent with authors who describe the artificial lakes of Yamoussoukro as eutrophic [21,23,54,55]. Human activities within the watershed and surrounding areas have led to this phenomenon, as the lakes receive significant amounts of pollutants, including erosion products, domestic waste, oil, and untreated sewage [54].

Then, bacterial isolates were identified as members of the *Acinetobacter*, *Citrobacter*, *Escherichia*, *Enterobacter*, *Pseudomonas*, *Kluyvera*, *Leclercia*, *Edwardsiella*, and *Proteus* genera. These isolates were dominated by *E. coli* at the species level in terms of relative abundance, but the genetic variability was more important in the genus *Acinetobacter.* About seven species of *Acinetobacter* were identified, including *Acinetobacter baumanii*, *A. junii*, *A. lactucae*, *A. pittii*, *A. soli*, *A. vivianii*, and *A. venetianus*. The presence of pathogenic bacteria in water systems and lettuce has been documented in numerous studies, with the incidence of such bacteria most often associated with human infections [15,56,57,58,59]. Additionally, some species have been linked to issues in aquaculture [60]. The identification of Gram-negative bacteria from both Yamoussoukro Lake and surrounding lettuce samples highlights the potential public health risks associated with environmental and food contamination.

The impact of the lake’s physicochemical parameters on the bacterial community (diversity and abundance) was assessed using Pearson correlation. Both positive and negative correlations were obtained; however, they were significant for only three parameters overall. Specifically, dissolved oxygen emerged as a determinant for bacterial diversity, showing a strong negative correlation. This result aligns with Anoman et al., 2019 [21]. As a fundamental and often limiting resource, dissolved oxygen is essential for the respiration of the vast majority of aquatic organisms, which includes a broad spectrum of bacterial species [61]. Obligate aerobes require high oxygen concentrations for optimal growth, while microaerophiles thrive in much lower concentrations. Facultative anaerobes can use oxygen if available but can also grow without it, whereas aerotolerant anaerobes do not use oxygen for growth but can survive in its presence [62]. This would impose selective pressure, where even marginal decreases in dissolved oxygen levels would be associated with a drastic reduction in bacterial diversity within the lake ecosystem. Furthermore, bacterial abundance was found to be significantly and positively correlated with both turbidity and pH. Studies have explicitly shown that increased concentrations of suspended solids can significantly enhance bacterial production and abundance [63,64]. High turbidity is often associated with indicators of water quality degradation, such as an increase in organic matter load, soil erosion, or dense algal blooms, all of which provide substrates and conditions conducive to bacterial proliferation [48]. Similar to turbidity, a high pH leads to an increase in biodegradable organic matter in the water, especially during the dry season, which favors the growth of microbial populations [61].

The genus *Acinetobacter* comprises a heterogeneous group of non-fermentative Gram-negative bacteria. They have recently become a focus of attention for scientists and clinicians, in terms of both their fundamental biological properties and their pathogenic potential [65]. In this study, *Acinetobacter soli* was the most prevalent of the lettuce isolates (>88%), contrasting with previous reports that identified *A. calcoaceticus* and *A. johnsonii* as the most common [66] *A. soli* was recently associated with invasive infection in Japan [67]. In the lakes, *Acinetobacter* species were more diverse, with *A. baumanii*, *A. vivianii*, *A. venetianus, A. junii*, and *A. lactucae* being identified. This result is in line with several previous studies and further highlights water as a reservoir of *Acinetobacter* species. However, data showing the existence of these species in aquatic environments is not a novel phenomenon [68,69,70]. Nevertheless, this finding is a matter of concern, particularly given the role of species such as *A. baumanii* in causing a significant number of human infections and, consequently, representing a public health risk [65]. *Acinetobacter* spp. have also been identified as a significant concern in terms of antibiotic resistance [66,67,69,70]. Resistances observed in this study were only to ceftazidime (82.35%), and no EBSL producers were detected. Thus, the mechanism of resistance observed was likely due to the production of chromosomal enzyme *AmpC* cephalosporinases, which confer resistance to broad-spectrum cephalosporins [71]. Moreover, the use of media containing ceftazidime might have facilitated the isolation of resistant strains.

It was expected that an important number of species from the order *Enterobacterales* would be present in both areas, given that these microorganisms constitute the largest Gram-negative family [3,58]. In the lake sample, the isolated species included *Escherichia coli*, *Citrobacter werkmanii, C. freundii*, *Edwardsiella tarda*, and *Proteus mirabilis*. While in the lettuce sample, the species included *C. werkmanii, C. cronae*, *E. coli*, *Enterobacter bugandensis*, *Enterobacter sichuanensis*, *Kluyvera georgiana*, and *Leclercia* spp. The presence of *E. coli* in the Yamoussoukro lakes and on the vegetables was previously evaluated, as well as antibiotic resistance to some antibiotics [21,22], lending support to the current findings. However, we reported the presence of other species from the order *Enterobacterales*, which increase the concern. In Iran, a foodborne outbreak was attributed to the bacterium *Citrobacter freundii*, with environmental factors identified as a contributing element in the contamination [72]. A further report has identified *Edwardsiella tarda* as a significant cause of concern, particularly in relation to its potential to cause severe food- and waterborne infections, with a high mortality rate among patients with underlying liver cirrhosis [73]. Thus, the order *Enterobacterales* encompasses both waterborne and foodborne species, reflecting the elevated level of contamination observed in these environments [74].

Moreover, the majority of antibiotic resistance observed in this study was confined to the order *Enterobacterales*. *Citrobacter* spp. exhibited resistance to cefoxitin (90.9%) and ampicillin (81.8%), and *Enterobacter* spp. demonstrated resistance to cefoxitin (75%), as they are naturally resistant to these antibiotics [75]. However, resistance to imipenem was also observed in 45.45% of *Citrobacter* spp. and 50% of *Enterobacter* spp. Additionally, imipenem resistance was observed in multiresistant strains from these genera. These findings are in accordance with those reported by Duran-Bedolla et al. [76]. Resistance to imipenem is frequently an indicator of carbapenemase multiresistance [77]. Concomitantly, *Kluyvera georgiana* also exhibited resistance to imipenem (50%), which aligns with recent studies describing its acquired resistance to carbapenems [78,79]. Therefore, further testing is required to confirm the presence of this multiresistance in the isolate in question.

The *E. coli* isolates demonstrated resistance to the majority of antibiotics tested, with a particularly high prevalence to ampicillin (90.9%), cefepime (72.72%), cefotaxime (68.18%), and aztreonam (63.63%). These findings align with those of Anokyewaa Appau et al. [80] in Ghana. The potential role of water as a reservoir for pathogenic *E. coli* has been documented in prior research [16,80,81]. It can reasonably be assumed that contamination of vegetables will occur during the process of irrigation. The isolate demonstrated multidrug resistance through the production of an extended-spectrum beta-lactamase, with *bla_CTX-M_* being the most dominant gene. Additionally, the *bla_TEM_* gene was identified in certain strains (73.33%). Initially identified in the *E. coli* species, this gene primarily mediates chromosomal resistance [6], although it can also be carried by plasmids. In contrast, the *bla_CTX-M_* gene is plasmid-mediated [6]. This genomic location facilitated the dissemination of the gene in a variety of environments. Furthermore, plasmids carrying the *bla_CTX-M_* gene have the capacity to harbor resistance genes to additional antibiotic classes [82], thereby intensifying the threat and public health risk associated with this resistance.

*Pseudomonas* spp. comprises a diverse range of species that are ubiquitous in various environmental niches, including soil, water, plants, food, and sewage. The *Pseudomonas* strains isolated in this study include *P. aeruginosa*, *P. putida*, and *P. monteilii*. These organisms are opportunistic pathogens that can cause serious infections in people with a weakened immune system. Among these species, *P. aeruginosa* stands out the most and is frequently associated with nosocomial infections [83,84] and drinking water contamination [85,86]. However, *P. monteilii* and *P. putida* have been reported to be associated with infections in humans [87,88]. Furthermore, *Pseudomonas* spp. exhibits high resistance, limiting the number of remaining antibiotics that can be utilized in treatment. In this study, multiresistant strains were obtained with a MAR phenotype pattern of “FEP-CIP-IPM.” Like *Citrobacter* spp. and *Enterobacter* spp., it is possible that these isolates exhibit carbapenemase resistance. *Pseudomonas* spp. carbapenemase-resistant bacteria have been identified as a significant public health concern by the WHO [5,89]. Therefore, this mechanism should be assessed in isolated samples to determine its prevalence.

The MAR phenotypes compiled for all the isolates indicated a high degree of multiple antibiotic resistance, with a range of resistance to three to nine antibiotics. The most prevalent MARPs observed included “AMP-FEP-ATM-CTX” and “AMP-FEP-ATM-CTX-AMC-CAZ,” which collectively represented 22.22% of the MARPs observed in the isolates. This suggests that the combination of these antibiotics may prove ineffective in treating human or animal infections [90]. Additionally, the MARP “AMP-FEP-ATM-CTX” was identified in both lake and lettuce isolates. However, due to the limitations of phenotypic analysis, it is challenging to infer the transmission of resistance with the same degree of accuracy as genotyping. The potential health risks associated with the dissemination of antibiotic resistance in an environmental setting are estimated using MARI [37,91]. A MARI value of 0.2 was employed to differentiate between low and high health risks. A value exceeding 0.2 indicates that the multiresistant isolates likely originated from an environment with high contamination or the overuse of antibiotics [37,90,91]. In this study, the MARI values obtained, predominantly ≥0.2, indicate an overuse or misuse of antibiotics in the environmental context from which the samples were collected. This could potentially lead to an increased transmission of MDR isolates to humans via the consumption of contaminated vegetables or direct contact with lake waters.

The daily use of lake water for irrigation is very common for farmers (100% of the surveyed producers), although it represents a continuous influx of potential hazards [19]. The results of this study showed that the manmade lakes of Yamoussoukro are suitable for irrigation. However, for human health, it is important to consider the high level of turbidity, eutrophication, opportunistic pathogenic bacteria, and antibiotic resistance, as discussed above. Thus, the lakes become reservoirs of contaminants, creating a cycle in which irrigation perpetuates the contamination of the food system [56]. Simultaneously, the use of chicken manure as fertilizer (used by 100% of the surveyed producers), especially when untreated, is a well-documented source of foodborne pathogens and is associated with ESBL resistance [80,92,93]. Consequently, solutions for water and lettuce quality in the Yamoussoukro area must be integrated into the “One Health” concept, where environmental health impacts human health through the food supply. Although farmers understand the general concept of foodborne illness, their complete lack of awareness of antibiotic resistance means that they are inadvertently contributing to a major global health crisis through their practices. Given this observation, it would be beneficial to implement training programs on good agricultural and hygiene practices, as well as awareness campaigns on antibiotic resistance, for lettuce producers.

## 5. Conclusions

Gram-negative bacteria represent a significant source of antimicrobial resistance. The findings of this study provide critical insights into the potential public health risks associated with their presence in both Yamoussoukro’s lakes and the lettuce grown in the area. The identification of a wide range of bacterial genera, including known pathogens, coupled with a high prevalence of antibiotic resistance and a MARI exceeding the threshold, highlights an urgent threat to public health. The presence of ESBL-producing *E. coli* and other multidrug-resistant isolates further underscores the need for immediate monitoring and intervention.

Future research should focus on larger-scale investigations that encompass source tracking of resistance genes, in-depth genomic characterization of the isolates (whole genome sequencing), and a more detailed analysis of the impact of agricultural practices and lake water quality on the dissemination of antibiotic resistance. These efforts are crucial for developing effective surveillance and intervention strategies to mitigate the spread of antibiotic-resistant bacteria and protect public health in Côte d’Ivoire.

## Figures and Tables

**Table 1:** Primer sequences, amplicon size, and cycling conditions used for the detection of ESBL resistance genes.

Genes	Name of Primer	Primer Sequence (5′–3′)	Size (pb)	Cycling	References
*bla*_TEM_	TEM front P1	GCG GAA CCC CTA TTT G	1017	Initial denaturation at 95 °C for 2 min, followed by 30 cycles of denaturation at 95 °C for 30 s, annealing at 55 °C for 30 s, 72 °C for 1 min 30 s, and a final extension at 72 °C for 2 min	[39]
	TEM-C-R-ny	ACC AAT GCT TAA TCA GTG AG			
*bla*_SHV_	SHV OS5	TTA TCT CCC TGT TAG CCA CC	797	Initial denaturation at 95 °C for 2 min, followed by 30 cycles of denaturation at 95 °C for 30 s, annealing at 60 °C for 30 s, 72 °C for 1 min s, and a final extension at 72 °C for 2 min	[40]
	SHV OS6	GAT TTG CTG ATT TCG CTC GG			
*bla*_CTX-M_	F	ATG TGC AGY ACC AGT AAR GTK ATG GC	593	Initial denaturation at 95 °C for 2 min, followed by 30 cycles of denaturation at 95 °C for 30 s, annealing at 60 °C for 30 s, 72 °C for 45 s, and a final extension at 72 °C for 2 min	[41]
	R	TGG GTR AAR TAR GTS ACC AGA AYS AGC GG			

**Table 2:** Sociodemographic characteristics of market gardeners around four artificial lakes in Yamoussoukro.

Characteristics	Distribution (%)			
	Lake 1 (*n* = 12)	Lake 5 (*n* = 3)	Lake 6 (*n* = 20)	Lake 8 (*n* = 30)
Gender				
Men	100	66.67	55	46.67
Women	0	33.33	45	53.33
Nationality				
Ivorian	83	66.67	55	100
Not Ivorian	17	33.33	45	0
Age				
<30 years	41.67	0	45	36.67
30 and 45 years	58.33	100	50	63.33
>45 years	0	0	5	0
Study level				
Illiterate	58.33	100	100	60
Primary	41.67	0	0	40
Experience in the sector				
<5 years	25	0	0	16.67
5 and 10 years	16.67	33.33	45	36.67
10 and 15 years	0	66.67	45	30
15 and 20 years	0	0	0	3.33
>20 years	58.33	0	10	13.33

**Table 3:** Physicochemical properties of lakes prospected in Yamoussoukro and their statistical significance.

Lake Properties	Lake 1	Lake 5	Lake 6	Lake 8	*p*-Value
NO_3_^−^ (mg/L)	8.86 ± 7.66 ^a^	1.28 ± 0.16 ^a^	14.81 ± 11.82 ^a^	4.74 ± 3.01 ^a^	0.199
NO_2_^−^ (mg/L)	2 ± 1.73 ^a^	0.29 ± 0.04 ^a^	3.34 ± 2.67 ^a^	1.07 ± 0.68 ^a^	0.199
PO_4_^3−^ (mg/L)	1.22 ± 1 ^a^	0.18 ± 0.05 ^a^	1.06 ± 0.1 ^a^	0.3 ± 0.23 ^a^	0.088
TDS (mg/L)	1.44 ± 0.49 ^b^	2.25 ± 0.06 ^a^	2.17 ± 0.07 ^a^	2.02 ± 0.09 ^ab^	<0.05
Conductivity (µS/cm)	2.41 ± 0.82 ^b^	3.77 ± 0.1 ^a^	3.67 ± 0.02 ^a^	3.31 ± 0.12 ^ab^	<0.05
Dissolved oxygen (mgO_2_/L)	5.07 ± 0.9 ^a^	5.87 ± 1.19 ^a^	5.43 ± 1.36 ^a^	4.47 ± 0.35 ^a^	0.44
NH_4_^+^ (mg/L)	0.65 ± 0.41 ^c^	11.82 ± 1.2 ^a^	10.45 ± 9.91 ^b^	5.43 ± 6.69 ^c^	<0.001
HCO_3_^−^ (mg/L)	142 ± 50.2 ^b^	334 ± 20.9 ^a^	310 ± 25.5 ^a^	259 ± 26.9 ^a^	<0.001
Ca_2_^+^ (mg/L)	0.41 ± 0.04 ^c^	0.66 ± 0.05 ^a^	0.67 ± 0.02 ^a^	0.55 ± 0.05 ^b^	<0.001
TH (mg/L)	0.54 ± 0.13 ^b^	0.81 ± 0.05 ^a^	0.9 ± 0.06 ^a^	0.76 ± 0.08 ^ab^	<0.01
Mg_2_^+^ (mg/L)	0.13 ± 0.1 ^a^	0.14 ± 0.04 ^a^	0.23 ± 0.05 ^a^	0.21 ± 0.11 ^a^	0.409
Turbidity (NTU)	7 ± 5.26 ^c^	95.67 ± 6.45 ^b^	107.63 ± 17.61 ^b^	216.67 ± 52.08 ^a^	<0.001
pH	5.96 ± 0.4 ^c^	7.32 ± 0.15 ^b^	7.35 ± 0.4 ^b^	8.47 ± 0.55 ^a^	<0.001
Temperature (°C)	22.83 ± 0.55 ^b^	25.83 ± 0.32 ^a^	24.83 ± 0.68 ^a^	25.2 ± 0.61 ^a^	<0.001
Redox potential (mV)	43 ± 24.52 ^b^	43 ± 9.17 ^b^	45 ± 24.58 ^b^	114 ± 34.64 ^a^	<0.05

Values represent means ± standard errors. Lake properties are abbreviated as follows: nitrate (NO_3_^−^), nitrite (NO_2_^−^), phosphate (PO_4_^3−^), ammonium (NH_4_^+^), bicarbonate (HCO_3_^−^), calcium (Ca_2_^+^), magnesium (Mg_2_^+^), total dissolved solids (TDS), total hardness (TH), and pH (hydrogen potential). The *p*-value represents the probability of error associated with Tukey’s post hoc test. Different letters (a, b, c) on the average values indicate statistically significant differences between the lakes according to Tukey’s post hoc test.

**Table 4:** Pearson correlation coefficients among bacterial diversity index, abundance, and surveyed lake chemical properties.

Parameters	Shannon Diversity Index	Abundance
NO_3_^−^ (mg/L)	0.14	0.025
NO_2_^−^ (mg/L)	0.14	0.025
PO_4_^3−^ (mg/L)	0.09	−0.461
TDS (mg/L)	−0.39	0.58
Conductivity (µS/cm)	−0.45	0.542
Dissolved oxygen (mgO_2_/L)	−0.99 **	−0.48
NH_4_^+^ (mg/L)	−0.64	0.33
HCO_3_^−^ (mg/L)	−0.49	0.494
Ca_2_^+^ (mg/L)	−0.52	0.46
TH (mg/L)	−0.31	0.63
Mg_2_^+^ (mg/L)	0.4	0.85
Turbidity (NTU)	0.48	0.962 *
pH	0.35	0.95 *
Temperature (°C)	−0.30	0.61
Redox potential (mV)	0.81	0.78

Statistically significant correlation results are indicated by asterisks, and the thresholds for correlation strength classifications are as follows: * *p* < 0.05, moderate correlation; ** *p* < 0.01, strong correlation.

**Table 5:** Patterns of MAR phenotypes and MAR index of the isolates from lake water and lettuce samples.

Isolation Source	MAR Phenotype	No. of Antibiotics	No. of Isolates	No. of Antibiotics Tested	MARI
Lake water	*E. coli*				
	AMP-IPM-CIP	3	1	11	0.27
	AMP-FEP-ATM-CTX	4	4	11	0.36
	AMP-FEP-CIP-ATM-CTX-AMC-CAZ	7	1	11	0.63
	AMP-FEP-ATM-CTX-AMC-CAZ	6	4	11	0.54
	AMP-CN-FEP-CIP-ATM-CTX-AMC	7	1	11	0.63
	*Pseudomonas* spp.				
	FEP-IPM-CIP	3	2	7	0.27
Lettuce	*E. coli*				
	AMP-CN-CIP-C	4	1	11	0.36
	AMP-FEP-ATM-CTX-AMC-CAZ	6	1	11	0.54
	AMP-CN-FEP-CIP-FOX-ATM-CTX-AMC-CAZ	9	1	11	0.81
	AMP-CN-FEP-FOX-ATM-CTX-AMC-CAZ	8	1	11	0.72
	AMP-CN-FEP-CIP-ATM-CTX-AMC-CAZ	8	1	11	0.72
	AMP-CN-FEP-ATM-CTX-CAZ	6	1	11	0.54
	*Citrobacter* spp.				
	AMP-IPM-FOX	3	1	11	0.27
	AMP-IPM-FEP-FOX	4	1	11	0.36
	AMP-IPM-FEP-CIP-FOX	5	1	11	0.45
	AMP-IPM-CIP-FOX	4	1	11	0.36
	AMP-IPM-CIP-FOX-ATM-C	6	1	11	0.54
	*Enterobacter* spp.				
	AMP-IPM-FOX-AMC	4	2	11	0.36

AMP: ampicillin; CN: gentamicin; IPM: imipenem; FEP: cefepime; CIP: ciprofloxacin; FOX: cefoxitin; ATM: aztreonam; CTX: cefotaxime; C: chloramphenicol; AMC: amoxicillin/clavulanic acid; CAZ: ceftazidime.

## Data Availability

The original contributions presented in this study are included in the article/Supplementary Materials. Further inquiries can be directed to the corresponding authors.

## Supplementary Materials

The following supporting information can be downloaded at https://www.mdpi.com/article/10.3390/microorganisms13091997/s1: Figure S1. Antibiogram of one resistant isolate. Table S1. Breakpoint used for the interpretation of the inhibition value. Table S2. Isolates analyzed in this study and their accession numbers for the 16S rDNA gene.
